# Influences of Orally Taken Carotenoid-Rich Curly Kale Extract on Collagen I/Elastin Index of the Skin

**DOI:** 10.3390/nu9070775

**Published:** 2017-07-19

**Authors:** Martina C. Meinke, Ceylan K. Nowbary, Sabine Schanzer, Henning Vollert, Jürgen Lademann, Maxim E. Darvin

**Affiliations:** 1 Charité-Universitätsmedizin Berlin, Corporate Member of Freie Universität Berlin, Humboldt-Universität zu Berlin, and Berlin Institute of Health, Center of Experimental and Applied Cutaneous Physiology (CCP), Department of Dermatology, Charité-Universitätsmedizin Berlin, 10117 Berlin, Germany; ceylan.nowbary@charite.de (C.K.N.); sabine.schanzer@charite.de (S.S.); juergen.lademann@charite.de (J.L.); maxim.darvin@charite.de (M.E.D.); 2 Bioactive Food GmbH, Am Ihlsee 36a, 23795 Bad Segeberg, Germany; henning_vollert@t-online.de

**Keywords:** skin aging, two photon tomography, reflectance spectroscopy, noninvasive methods, auto fluorescence, second harmonic generation, nutrition

## Abstract

Two differently designed, spatially resolved reflectance spectroscopy-based scanners and two-photon tomography were used for noninvasive in vivo determination of cutaneous carotenoids, and collagen I/elastin aging index of dermis, respectively, in the skin of 29 healthy female volunteers between 40 and 56 years of age. The volunteers received a supplement in the form of a carotenoid-rich natural curly kale extract containing 1650 µg of carotenoids in total (three capsules of 550 µg), once a day. Measurements were taken before, after 5 months and after 10 months of daily supplementation. The results showed significantly increased values for the cutaneous carotenoids and the collagen I/elastin aging index of dermis 5 and 10 months after the beginning of the study. The obtained results show that a natural carotenoid-rich extract could prevent the aging-related collagen I degradation in the dermis and improve the extracellular matrix.

## 1. Introduction

Nutrition rich in fruit and vegetables is beneficial to human health and wellbeing [1,2,3,4]. Secondary plant components, such as antioxidants [5], protect the cells and connective tissue against the development of oxidative stress, which is related to several pathological consequences, e.g., neurological [6], cardio-vascular [7], dermatological diseases [8] or aging [9]. Oxidative stress is induced by various factors, inter alia, solar radiation, smoking, alcohol consumption, lack of sleep, intensive physical activities and psychological stress [10,11,12,13,14,15,16,17,18,19,20], and therefore cannot be avoided in daily life. Oxidative stress is characterized by the generation of large amounts of reactive oxygen species (ROS), whose action is responsible for the development of premature skin aging and photo aging [7,21]. To counteract this effect, antioxidants should be available in sufficient concentration and composition to keep the balance and avoid tissue damage [22]. The majority of the essential antioxidants, including carotenoids, have to be consumed via dietary sources. The uptake and bioavailability of carotenoids can be measured in vivo by resonance Raman and reflectance spectroscopy [23,24]. Furthermore, it could be demonstrated that skin with a high amount of the carotenoid lycopene is less rough and therefore looks younger [21]. The physiologically relevant composition and concentration of antioxidants is important, as at very high concentrations, antioxidants might reverse their properties and become pro-oxidant [25,26]. This process is intricate and depends on many factors including oxygen tension and further antioxidants such as α-tocopherol in the tissue [27,28]. Taking this into account, a promising anti-aging strategy could be the physiologically relevant enhancement of carotenoids and other antioxidants in the tissue by nutrition and/or supplements [22].

The beneficial effects of such application on the skin status were studied based on skin parameters such as furrows and wrinkles, elasticity and skin hydration [29]. To understand such effects, more than roughness and wrinkle volume should be measured. The dermal proteins collagen I and elastin are related to the skin age and could serve as reliable parameters of the anti-aging effect of nutrition or a supplement [30,31]. Various studies have shown that the collagen I/elastin ratio, as well as the collagen I and elastin structures, change with age [32,33,34,35]. The elasticity of the skin increases with atrophy of the collagen I fiber bundles, the related flattening of the rete ridges, and the thickening of the dermal elastic fibers. Previously, biopsies had to be taken, but nowadays, noninvasive two-photon tomography is available to measure the collagen I by second harmonic generation (SHG) and the elastin by auto fluorescence (AF) signals in vivo in the dermis [36,37]. The SHG to AF aging index of dermis (SAAID) is a noninvasive objective parameter for determining the collagen I status in the dermis, which can be used for the description of the intrinsic and extrinsic aging on various skin areas in vivo [38,39], effect of skin protection [40], as well as efficacy of wound healing [41] and identification of disease affected skin [42,43]. The collagen I/elastin index can be quantified with the SAAID, which is defined according to [42]:

SAAID = (SHG − AF)/(SHG + AF)
(1)

SAAID-scoring is a powerful method for analyzing the dermis dependent on AF and SHG signal intensities [43]. Other dermal collagens of types IV, VII and XVII are also known to reduce with age [44], but it is impossible to determine their concentrations in vivo in the skin. This can only be done by applying special algorithms [45].

The hypothesis is that systemically administered carotenoids will increase the collagen I synthesis or at least prevent its decrease, which usually occurs due to oxidative stress during our life, and results in skin aging. In the presented in vivo study, two different cutaneous carotenoid sensors, which were recently developed based on spatially resolved reflectance spectroscopy (SRRS), were applied to monitor the skin carotenoids noninvasively [46]. To increase the skin carotenoids, a vegetable curly kale extract rich in carotenoids, in comparison to a placebo, was systemically administered over a time period of 10 months. The curly kale extract was selected because previous studies had shown its positive effects on the skin in terms of bioavailability and radical scavenging activity [47,48]. The SAAID was measured as an objective skin aging parameter. The measurements were performed before, after 5, and after 10 months of intake of the carotenoid-rich curly kale supplement.

## 2. Material and Methods

### 2.1. Subjects

29 healthy female volunteers (10 smokers, 19 non-smokers) aged between 40 and 56 years (mean 49.2 years) of skin type II according to the Fitzpatrick classification [49] were enrolled in the study. 

Exclusion criteria were subjects with skin diseases, admission to an institution due to an administrative or judicial order (according to § 29 MPG), known drug addiction or alcoholism, and expectant or lactating women. The investigations were carried out in accordance with the ethical guidelines of the Declaration of Helsinki and had been approved by the local Ethics Committee prior to starting the study (EA1/229/14). All volunteers gave their informed written consent.

### 2.2. Supplements

During the uptake study, the volunteers ingested three capsules of a curly kale extract, hereinafter called verum, once a day (provided by BioActive Food, Bad Segeberg, Germany). One capsule contained the following substances: 430 µg lutein, 70 µg beta-carotene, 30 µg lycopene, 20 µg zeaxanthin, i.e., 550 µg carotenoids, analyzed independently by Bioanalyt GmbH (Potsdam, Germany). As a control, a placebo capsule containing an olive oil without carotenoids was given. The content of the capsules was measured with resonance Raman spectroscopy and the placebo did not show any carotenoid values.

### 2.3. Study Protocol

The volunteers were randomly divided into placebo (*N* = 15) and verum groups (*N* = 14).

The measurements were performed on the palm, the inner forearm and the cheek. Carotenoids can be sensitively determined only at the palm as the stratum corneum is sufficiently thick there and the measurements are not influenced by blood and melanin. The SAAID was measured on the cheek and the inner forearm to have one sun exposed and one sun protected area.

### 2.4. Carotenoid Determination

For the investigations, two different sensors were applied. One optical sensor was based on multiple spatially resolved reflectance spectroscopy (MSRRS). This sensor is commercially available under the tradename biozoom (model Biozoom Portable, Kassel, Germany). It uses several differently positioned light sources and light detectors, exhibiting different distances between light source and detector and different angles for irradiation and detection, and has been described in detail elsewhere [23,46]. Briefly, the LED light sources cover a spectral range from approximately 350 nm to 1000 nm in wavelength in 16 steps, providing 118 light emitters. The sensor picks up the backscattered light at a total number of 152 light sensitive areas. This combination results in almost 18,000 raw data values, which are picked up several times during one measurement at a sensor surface area of 20 mm by 20 mm.

The second carotenoid sensor was a CaroLED sensor (Laser-und Medizin-Technologie Berlin, LMTB, Germany). The underlying measuring principle of CaroLED is based on SRRS, and was recently described by Meinke et al. [46]. Briefly, five light emitting diodes (LED) of different wavelengths in the visible and near infrared region illuminate the skin through a measurement window. While light propagates through the individual skin layers, and the spectral signature of carotenoids and of other skin constituents is obtained [50]. Four photodiodes, each of which is placed at a different lateral distance to the illumination site, measure the spectral signature at different depth sensitivities.

The measurements using both sensors were performed five times on the same palm area (thenar eminence) of the right hand for all subjects, and mean values were calculated for each subject and measurement method before the statistical analysis was applied. Because of the different initial values, the data were normalized to the initial values, dividing the values from visit after 5 and 10 months by the initial values.

Both sensors were calibrated before starting the study using resonance Raman spectroscopy on the palm of healthy volunteers in vivo using a resonant excitation at 488 nm [46].

### 2.5. Collagen I/Elastin Index SAAID

The collagen I and elastin structure of the skin can be characterized by the second harmonic generation (SHG) to autofluorescence (AF) aging index of dermis (SAAID). The SAAID can be determined in vivo using two-photon microscopy by calculation of the corresponding AF and SHG image intensities [51,52]. The SAAID is defined as described in Equation (1). In the dermis, the AF is mainly determined by the elastin concentration, while the SHG is determined by the collagen I concentration. In this nomenclature, the SAAID decreases with photo aging, approaching its minimal value-1 when the collagen is completely replaced by elastic fibers.

The investigations were carried out using a two-photon tomograph (JenLab GmbH, Jena, Germany) equipped with a femtosecond titanium sapphire laser (Mai Tai XF, Spectra-Physics, Santa Clara, CA, USA). The laser was operated at 760 nm and generated 100 fs pulses at a repetition rate of 80 MHz. For studying the structure of the dermis at different depths of the skin, sensitive photo multipliers were applied. The radiation collected from different depths was averaged over slices of 200 × 200 µm^2^. The acquisition time was equal to 7 s for one slice, thus ensuring a satisfactory measurement stability and picture quality [41].

During the measurements, the laser focus was moved from the skin surface into deeper parts of the skin at 10 µm increments. The maximum measuring depth into the skin was 120 µm. For the whole depth, the SAAID was calculated and the profiles were normalized to the onset of the SHG signal. Four values after the SHG’s onset were considered within the mean values to ensure data from the upper part of papillary dermis below the basal layer. The utilized two-photon tomograph has been described in detail elsewhere [40].

The measurements were performed in vivo at the lower inner arm and the cheek. For all subjects, the area at the forearm was selected at 30 cm from the middle finger tip. The cheek area was selected, as it had the best coupling possibility with the optical measuring head on the line between nose and ear.

### 2.6. Statistics

The data were statically analyzed using SPSS for Windows (IBM^®^ SPSS^®^ Statistics, version 19, Inc., Chicago, IL, USA). The data were analyzed to a normal distribution using explorative data analysis. An unpaired *t*-test was applied to determine differences in the mean values between the groups, and a paired *t*-test for changes within one group. For the correlation, the Pearson correlation coefficient was calculated. Values of *p* ≤ 0.05 were considered to indicate a significant difference.

## 3. Results

Although 34 volunteers were enrolled, only 29 completed the study successfully. 5 dropouts occurred, 3 in the verum group and 2 in the placebo group. 2 volunteers quit the study due to problems with the stomach, and 3 did not come to the investigation after the 3rd visit and therefore were not replaced. All 29 volunteers underwent the carotenoid measurements and the two-photon tomography.

### 3.1. Carotenoids

**Figure 1:**
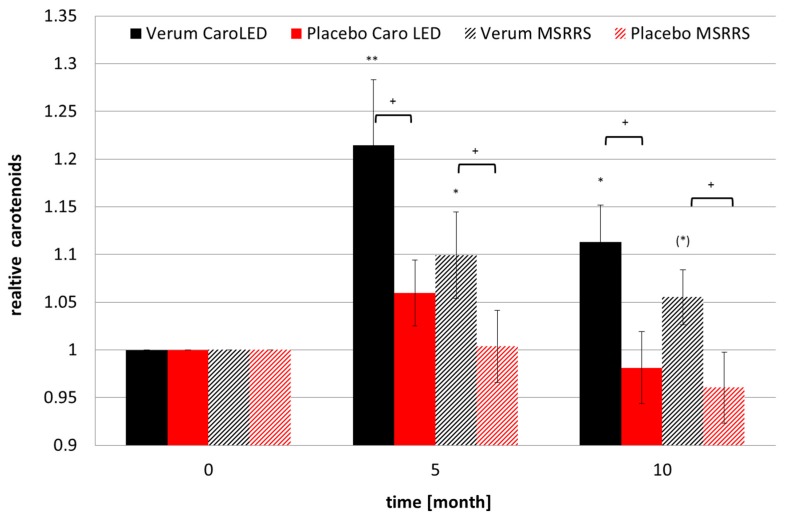
Carotenoid values relative to the initial values measured by two different carotenoid sensors obtained in the palm (mean ± SEM). + *p* < 0.05 between placebo and verum; (*) *p* < 0.1, * *p* < 0.05, ** *p* < 0.01 to initial value. *N* = 15 for placebo and *N* = 14 for verum.

The group-averaged cutaneous carotenoid concentrations measured using both carotenoid sensors are shown in Figure 1. The data are presented normalized to the initial value because the individual values show a high variation. The carotenoid values of the verum group measured with both devices increased significantly within the first 5 months, in which the volunteers ingested the carotenoid-rich extract, and decreased again over the next 5 months. But the values of the verum group after 10 months were still significantly higher than the initial value. The placebo values measured by CaroLED also increased and decreased, but the changes were not significant. The carotenoid concentration in the placebo group remained unchanged for the first 5 months and showed an insignificant decrease after 10 months of supplementation. 

### 3.2. SAAID

**Figure 2:**
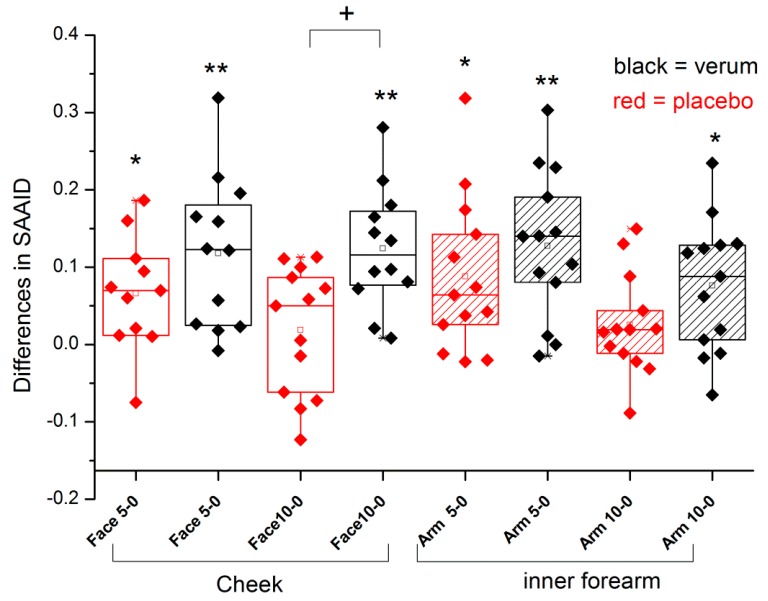
Box plot of the differences of SAAID to the initial values measured at the cheek and inner forearm. + *p* < 0.05 between placebo and verum; * *p* < 0.05, ** *p* < 0.01 to initial value. The box presents 50% of the data. The bottom and top of the box are always the first and third quartiles and the solid line box is always the second quartile, the median.

The SAAID was measured at the inner forearm and at the cheek. The differences from the initial values are shown in Figure 2. After 5 months, all SAAID values had increased significantly (*p* < 0.05 for placebo and *p* < 0.01 for verum), but after 10 months, the increase had remained significant only for the verum group. The verum group consistently showed higher values than the placebo group, and the difference between the two groups was significant for the cheek after 10 months (*p* < 0.05). 

### 3.3. Correlations

If the hypothesis is correct that the SAAID increases during the intake of carotenoids, a correlation of these two values is possible. Therefore, the Pearson correlation coefficient of the SAAID values and the carotenoid values was calculated (Table 1) for all subjects from whom at least one SAAID value (cheek or arm) was obtained for all visits (max. *n_subjects_* = 27, maximum measured values *N* = 81). As a positive check, the correlation between the two carotenoid values was determined, as well. As expected, the two carotenoid values have a highly significant positive correlation. Furthermore, the two SAAID values show a moderately positive correlation, which is highly significant. The correlations between the carotenoid and the SAAID values are between slightly and moderately positive with an *r* of about 0.3, and are significant. 

## 4. Discussion

Human nutrition is subject to seasonal variations, and the intake of secondary plant substances such as carotenoids and other antioxidants is related to it. From former investigations, it is known that the carotenoid status in humans is enhanced in the summer and autumn months due to the availability of regional fresh and ripe fruits and vegetables. This has been referred to as “seasonal increase” [53]. In the current study, the first measurements were performed in autumn and the second ones in the springtime. The last visit took place at the end of summer and mainly in autumn. This could explain the observed changes in the placebo group. Furthermore, infections, which occur very often in the wintertime, additionally decreased the carotenoid values in the skin [53].

As expected, the skin carotenoid values increased by 10 to 20% in the group that received the carotenoid-rich curly kale extract. This increase is below the data obtained in former studies because the dose of carotenoids in this long-term study was lower compared to former studies [47,48]. Nevertheless, the obtained enhancement was significantly higher than that of the placebo.

The investigations performed using two-photon tomography have shown that the SAAID also increased at the forearm and cheek in the placebo group. However, the enhancement of the SAAID of the verum group was always higher than the placebo. This indicates new collagen I production or a reduced thickening of the dermal elastic fibers. So far, it has not been observed in vivo that nutrition or supplementation results in such an effect. The more pronounced differences at the solar-exposed cheek compared to the protected inner forearm indicate not only a protection by the antioxidants and less degradation in the verum group, but also an improvement of the extracellular matrix compared to the first visit. Exposure to solar radiation induces free radicals, which interact with the extracellular matrix determinants once a critical threshold is reached [40]. This effect is called photo aging or premature skin aging [54,55,56]. The radical scavenging activity of the utilized curly kale extract has already been shown in a former in vivo study using electron paramagnetic resonance spectroscopy [48]. The verum group showed a higher radical scavenging activity in the skin and, as a result, an enhanced protection against VIS/NIR irradiation.

Interestingly, a correlation between the carotenoids and the SAAID values was obtained. These findings also suggest that the significant increase of SAAID values after 10 months could be due to the intake of the carotenoid-rich extract. The tendency towards correlation between SAAID and carotenoids has previously been reported [40], but such a supplementation-induced effect has not been observed so far.

The long-term study indicates that anti-aging effects are not easy to observe because many other factors could also influence the values. The measurements are only snapshots during this long-term investigation. Nevertheless, the SAAID could serve as a reliable parameter for the determination of the skin aging status, which is not so sensitive to short-term changes.

The above-mentioned skin parameters were measured noninvasively and in vivo, and do not interfere with each other.

## 5. Conclusions

In the performed placebo-controlled in vivo study, it was demonstrated with two different optical scanners that oral supplementation of the carotenoid-rich natural curly kale extract (1650 µg of total carotenoids) once a day had caused a statistically significant increase of carotenoids in the skin 5 and 10 months after the beginning of the study. In the cheek and forearm skin areas, the collagen I/elastin ratio (SAAID value) increased significantly in both the verum and placebo groups, although it was higher in the verum group. After 10 months of supplementation, the SAAID value in the verum group had remained significantly higher. Thus, the results show that a natural extract containing a mixture of carotenoids at physiological concentrations could prevent skin aging and improve the extracellular matrix. Therefore, a healthy lifestyle including a diet rich in carotenoids is the best prevention strategy against premature skin aging.

## Figures and Tables

**Table 1:** Pearson correlation coefficient r with *p*-values and number of the SAAID and carotenoid values (*N*); * *p* < 0.05, ** *p* < 0.01.

	SAAID Cheek	SAAID	Carotenoids
		Forearm	CaroLED
SAAID forearm			
Correlation according to Pearson	0.504 **	1	
Significance	<0.001		
*N*	76	77	
Carotenoids CaroLED			
Correlation according to Pearson	0.279 *	0.295 **	1
Significance	0.014	0.008	
*N*	77	80	81
Carotenoids MSRRS			
Correlation according to Pearson	0.328 **	0.225 *	0.853 **
Significance	0.004	0.023	<0.001
*N*	77	80	81
